# A Case of Immediate Reduction of Severe Mitral Regurgitation After the Ablation of Atrial Flutter

**DOI:** 10.7759/cureus.80053

**Published:** 2025-03-04

**Authors:** Daoud Eldawud, Farzane Saeidifard, Ammar Y Abdulfattah, Zaid Nakadar, Tanuj Gupta, Martin Weinstock, Cristina A Mitre

**Affiliations:** 1 Department of Internal Medicine, State University of New York Downstate Medical Center, Brooklyn, USA; 2 Department of Cardiology, State University of New York Downstate Medical Center, Brooklyn, USA; 3 Department of Cardiology, Veterans Affairs New York Harbor Health Care, Brooklyn, USA

**Keywords:** atrial flutter, atrial flutter ablation, echocardiography, electrophysiology study, mitral regurgitation

## Abstract

Mitral regurgitation (MR) is a common valvular dysfunction often classified as primary or secondary, with the latter typically associated with left ventricular dysfunction or mitral annular dilation. A subset of MR is termed atrial functional MR, related to atrial fibrillation, but the relationship between atrial flutter and MR remains underexplored. This report describes the case of a 71-year-old man with severe MR and atrial flutter who experienced rapid improvement in MR severity following successful atrial flutter ablation and restoration of sinus rhythm. Initial echocardiography revealed severe left atrial dilation, moderate to severe eccentric MR, and moderate tricuspid regurgitation. Following ablation, MR severity significantly improved despite persistent left atrial dilation, with sustained improvement observed over two years alongside reverse remodeling of the left atrium. This case highlights the independent effect of atrial flutter on MR severity, separate from structural remodeling, and emphasizes the potential for rhythm control strategies to improve MR and avoid invasive valve interventions. It also raises important questions about the interplay between atrial arrhythmias and MR, underscoring the need for further studies to better understand atrial functional MR and its management.

## Introduction

Mitral regurgitation (MR) is one of the most prevalent types of valve dysfunction, and its prevalence is expected to rise over the next few decades [1]. MR is traditionally classified as primary, due to intrinsic valve disease involving the chordae tendinae, papillary muscle, and annulus, or secondary (functional) which involves structural abnormalities in the left ventricle (LV) or left atrium (LA) rather than intrinsic valve disease. Some studies suggest that functional MR is primarily due to LV dysfunction or mitral annular dilation while others postulate that functional MR is the result of atrial dilation that occurs during atrial fibrillation; this dilation prevents the mitral leaflets from coapting properly during systole [2,3]. The importance of this differentiation stems from the primary form of treatment involved. With the intrinsic defects of the mitral valve present in primary MR, surgery is often indicated, while in cases of secondary MR, LV dysfunction being the main issue, goal-directed medical therapy is often the first-line treatment. If MR is left untreated, it can lead to progressive LV dysfunction and heart failure [4] 

The association between atrial flutter and functional MR has been less discussed. MR can lead to atrial enlargement and dilation and increased atrial pressure, which can lead to arrhythmias such as atrial flutter. Inversely, the mechanical impact of atrial flutter itself can affect the mitral valve. Atrial flutter can cause abnormal mitral valve motion, including premature closure and systolic flutter of the mitral valve leaflets, exacerbating MR [5].

In contrast to functional MR caused by LV dysfunction or dilation, the prevalence of atrial functional MR due to atrial fibrillation/flutter is unknown, but several risk factors have been identified such as heart failure with preserved ejection fraction (HFpEF), older age, left atrial size, and female sex [2,6]. Among patients presenting for atrial fibrillation ablation, the prevalence of atrial functional MR has been reported to range from 3% to 15% [7-9]. The coexistence of HFpEF and atrial fibrillation may be associated with a higher prevalence of atrial functional MR [10]. In HFpEF, LA enlargement and dysfunction are common, contributing to increased mitral annular dimensions and impaired mitral valve coaptation, which can lead to atrial functional MR. Management of atrial functional MR in the context of HFpEF often includes rhythm control strategies [11]. In atrial functional MR, the posterior mitral leaflet becomes restricted in its motion due to being pulled in opposite directions, upward by the displaced annulus and downward by the papillary muscles, leading to improper valve closure and regurgitation explaining the eccentric regurgitant jet [7]. 

Several recent studies demonstrated a reduction in MR severity after successful atrial fibrillation ablation or direct cardioversion [7,12,13]. However, the reversibility of MR severity in relation to atrial flutter is not thoroughly understood. Another reason to focus on atrial functional MR is because it has a better prognosis compared to ventricular functional MR [14]. Hirji et al. found that patients with atrial functional MR had significantly higher survival rates and freedom from re-operation at five and 10 years compared to those with ventricular functional MR [15]. 

This report presents a case illustrating the rapid change in MR severity in a patient with atrial flutter after successful ablation and restoration of sinus rhythm.

## Case presentation

A 71-year-old man with a medical history of alcohol abuse, decompensated cirrhosis (previous admission for hepatic encephalopathy and small non-bleeding esophageal varices), sinus node dysfunction with a permanent pacemaker, paroxysmal atrial fibrillation, and mild aortic stenosis initially presented with an episode of coffee ground emesis and black tarry stool in the setting of alcohol intoxication. This was his first presentation with bleeding. 

He was hemodynamically stable and his vital signs on presentation were within normal limits. His initial labs were significant for hemoglobin of 14.1 g/dL, hematocrit of 41.9%, mean corpuscular volume of 82 fL, red blood cells 4.8 million cells/mcL, platelets 90 K/uL, international normalized ratio (INR) 1.5, total bilirubin 1.3, aspartate aminotransferase 58 U/L, alanine aminotransferase 43 U/L, and alkaline phosphatase 110 U/L (Table 1). He was admitted for observation and serial hemoglobin checks. On admission, apixaban for paroxysmal atrial fibrillation was held and he was placed on an intravenous omeprazole infusion, octreotide, and prophylactic ceftriaxone. On repeat labs, his hemoglobin was stable at 13.9 g/dL and he remained hemodynamically stable. He was evaluated by the Gastroenterology and Hepatology services and recommended an elective endoscopy on an outpatient basis, which was also preferred by the patient over an inpatient procedure.

**Table 1:** Initial significant laboratory values INR: international normalized ratio

Test	Patient values	Reference ranges
Hemoglobin	14.1 g/dL	13.5–17.5 g/dL (men)
Hematocrit	41.9%	41.0–53.0% (men)
Mean Corpuscular Volume (MCV)	82 fL	80–100 fL
Red Blood Cells (RBC)	4.8 million cells/mcL	4.7–6.1 million cells/mcL (men)
Platelets	90 K/uL	150-450 K/uL
INR	1.5	0.8–1.2
Total Bilirubin	1.3 mg/dL	0.1–1.2 mg/dL
Aspartate Aminotransferase (AST)	58 U/L	10–40 U/L
Alanine Aminotransferase (ALT)	43 U/L	7–56 U/L
Alkaline Phosphatase	110 U/L	44–147 U/L
Thyroid-Stimulating Hormone (TSH)	3.6 mIU/L	0.4–4.0 mIU/L
Free T4	1.2 ng/dL	0.8–1.8 ng/dL

**Figure 1:**
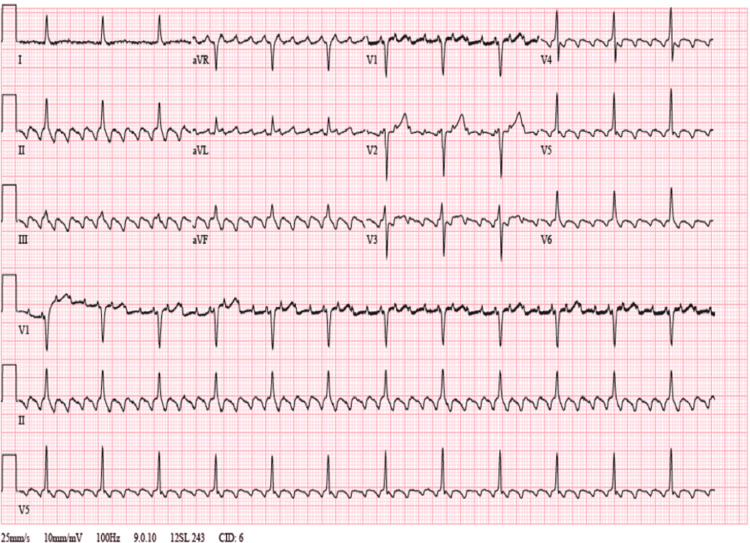
Initial electrocardiograph showing atrial flutter with 4:1 conduction at a ventricular rate of about 75 beats per minute. There are negatively directed sawtooth waves seen in the inferior leads II, III, and aVF, and positive waves are seen in lead V1.

His initial ECG during this admission showed a typical atrial flutter rhythm with 4:1 conduction at a rate of 75 beats per minute (Figure 1). Thyroid-stimulating hormone (TSH) level was 3.6 mIU/L and free T4 was 1.2 ng/dL (Table 1). 

**Figure 2:**
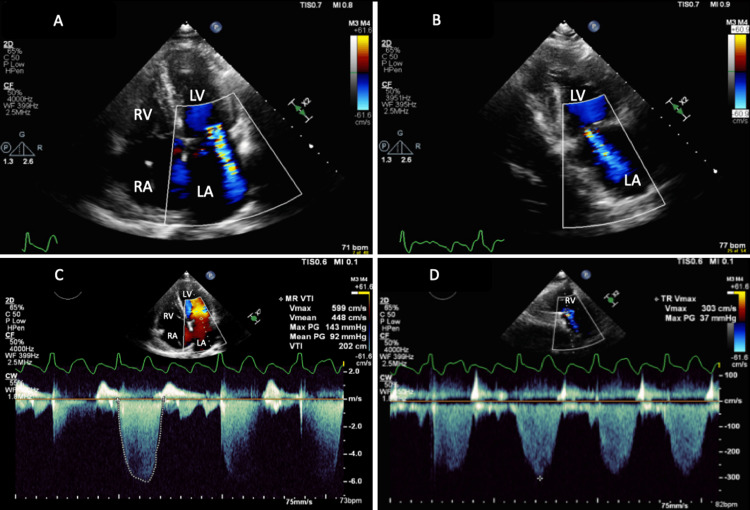
(A and B) TTE apical four-chamber view and two-chamber view with color Doppler, respectively, showing moderate to severe eccentric jet MR; (C) Apical four-chamber view showing the maximum velocity of MR at 599 cm/s by continuous wave Doppler during atrial flutter; (D) Parasternal long axis view with color Doppler showing moderate TR with TR Vmax 303 cm/s LA: left atrium; LV: left ventricle; RA: right atrium; RV: right ventricle; MR: mitral regurgitation; VTI: velocity time integral; Vmax: peak velocity; TR: tricuspid regurgitation; TTE: transthoracic echocardiography

His initial transthoracic echocardiogram (TTE) showed normal LV systolic function with normal wall motion and dimensions, severe LA dilation with an LA volume index (LAVI) of 59 ml/m², a calcified aortic valve with mild aortic stenosis and regurgitation, moderate to severe eccentric jet MR, moderate tricuspid regurgitation (TR), and no diastolic dysfunction. Figure 2 displays the color Doppler of MR in four- and two-chamber views, as well as the maximum MR velocity by continuous wave Doppler in the apical four-chamber view; TR velocity by continuous wave Doppler in the parasternal long-axis view is also shown. The patient was evaluated by the Electrophysiology service as he elected for an electrophysiology study and atrial flutter ablation. He underwent numerous ablations to the isthmus initially, which restored normal sinus rhythm. 

**Figure 3:**
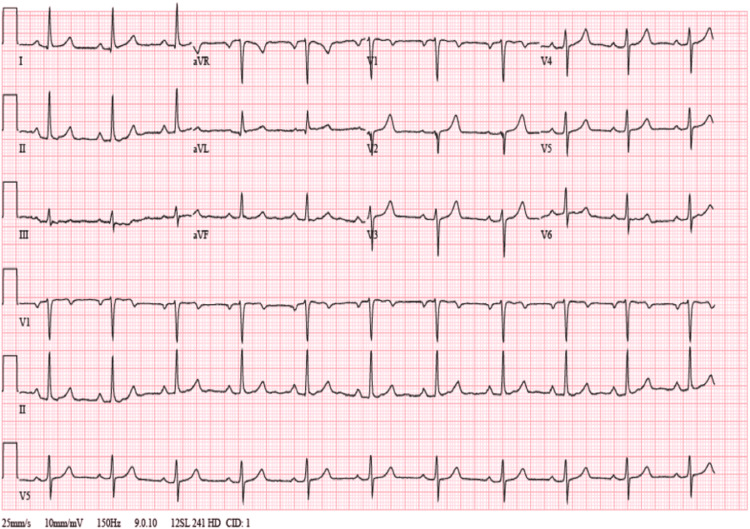
Electrocardiograph (ECG) at three-week follow-up showing normal sinus rhythm at 67 beats per minute

**Figure 4:**
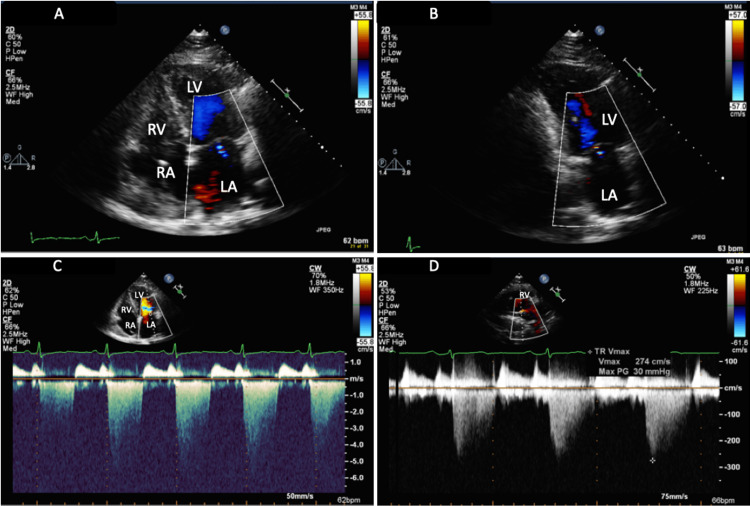
(A and B) TTE apical four-chamber view and two-chamber view with color Doppler, respectively, showing improved MR with reduced MR jet; (C) Apical four-chamber view showing improved maximum velocity of MR at around 450 cm/s by continuous wave Doppler in normal sinus rhythm; (D) Right ventricular inflow tract view with color Doppler showing improvement in TR with TR Vmax 274 cm/s LA: left atrium; LV: left ventricle; RA: right atrium; RV: right ventricle; TR: tricuspid regurgitation; Vmax: peak velocity; TTE: transthoracic echocardiography; MR: mitral regurgitation

Three weeks after the successful atrial flutter ablation, the patient was followed up in the electrophysiology clinic and an ECG revealed normal sinus rhythm (Figure 3). Using the same ultrasound system and sonographer, the patient underwent a repeat TTE which showed a normal ejection fraction of 55%, normal LV wall thickness, persistent severe LA dilation (LAVI 59 ml/m²), mild aortic stenosis with trace aortic insufficiency, a calcified mitral apparatus and improvement in both the MA and tricuspid regurgitation (Figure 4).

**Figure 5:**
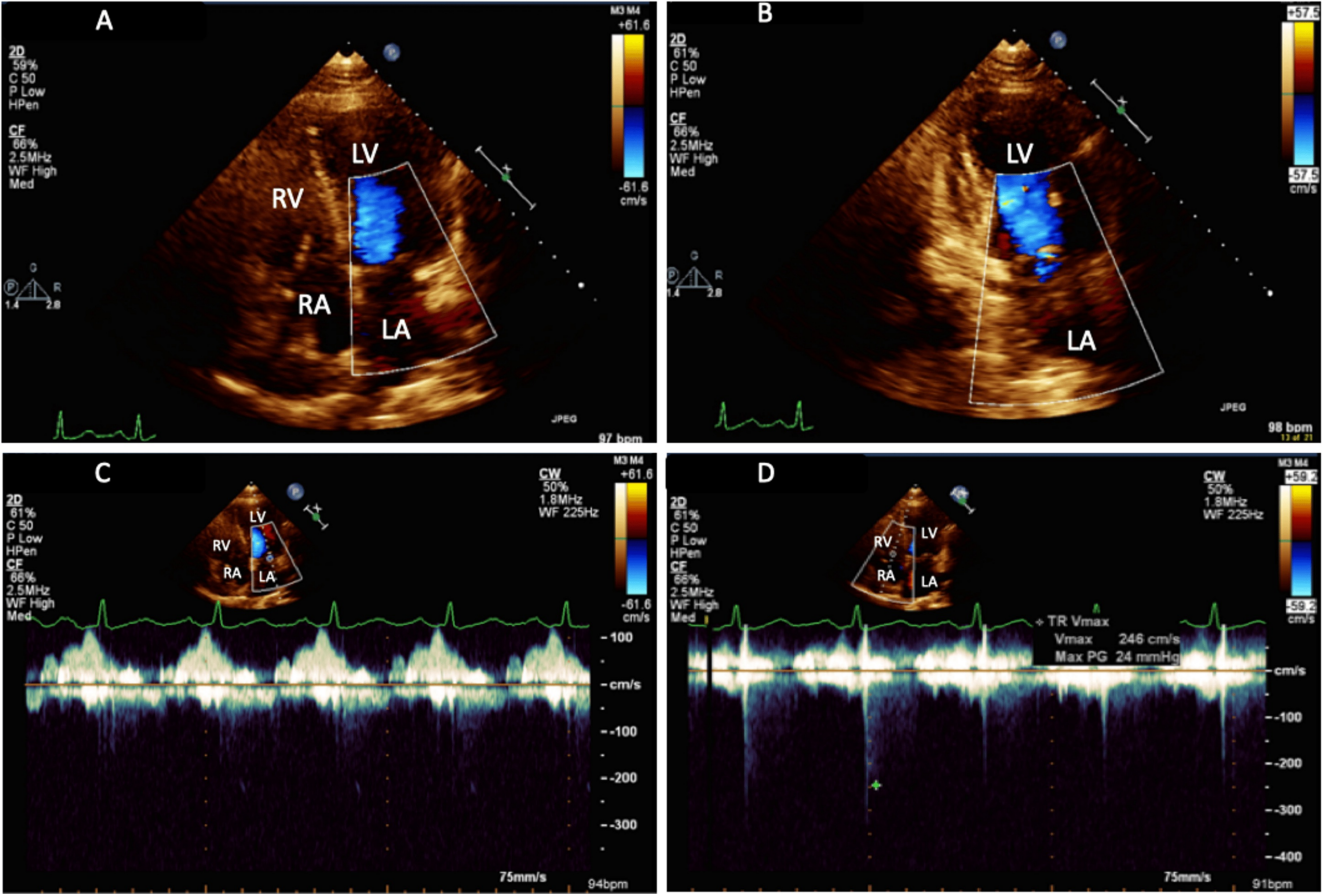
(A and B) TTE apical four-chamber view and two-chamber view with color Doppler, respectively, showing improved MR with reduced MR jet; (C) Apical four-chamber view showing trace MR by continuous wave Doppler in normal sinus rhythm; (D) Apical four-chamber view with color Doppler showing trace TR with TR Vmax 246 cm/s LA: left atrium; LV: left ventricle; RA: right atrium; RV: right ventricle; TR: tricuspid regurgitation; Vmax: peak velocity; TTE: transthoracic echocardiography; MR: mitral regurgitation

The patient remained in sinus rhythm without being placed on anti-arrhythmic medications with minimal symptoms during subsequent clinic visits. Two years later, his repeat TTE showed a normal LV systolic function. His LA size had significantly decreased, with LAVI of 41 ml/m² (mild to moderate LA dilation). TTE also showed a calcified mitral apparatus with only trace MR and trace TR (Figure 5).

The reduction in MR velocity from 599 cm/s to 450 cm/s, and ultimately to trace regurgitation, represents a significant hemodynamic improvement, as higher velocities indicate a greater pressure gradient across the mitral valve and more severe regurgitation. This reduction correlates with decreased volume overload on the LA and improved forward cardiac output, leading to alleviation of the patient’s shortness of breath. The increased velocity in MA reflects the hemodynamic consequence of regurgitant flow into the LA during systole, causing elevated LA pressure and volume. Upon mitral valve opening in diastole, the heightened pressure creates a steeper atrial-ventricular gradient, resulting in faster early filling and thus higher regurgitant velocity.

## Discussion

This case presents the change in the severity of MR after returning the heart to sinus rhythm from atrial flutter. After successful ablation of atrial flutter, there was a drastic reduction in the severity of MR before left atrium remodeling occurred. This report suggests the association of atrial flutter with MR as a type of atrial functional MR, which is not well described.

Atrial fibrillation-induced atrial dilatation leading to mitral annular dilatation is the proposed mechanism of atrial functional MR [16-19]. Annular dilation is defined as a systolic anteroposterior diameter of 3.4 cm and above or when the ratio of the systolic annular diameter to diastolic anterior leaflet length exceeds 1.3 cm [1]. It is also proposed that tethering of leaflets during atrial fibrillation can contribute to the severity of MR, which is usually seen in ventricular functional MR; however, its exact effect on MR is not yet fully understood [16-18]. A few studies using three-dimensional (3D) transesophageal echocardiogram have tried to clarify the mitral geometric changes in patients with atrial functional MR [1,19].

On the other hand, in atrial flutter, flutter waves have mechanical effects on mitral valve dynamics, and these waves can cause the mitral valve to open and close with each flutter wave during diastole, especially in patients with higher degrees of AV block. When there are long RR intervals, flutter waves may cause early valve closure before ventricular systole, and subsequent flutter waves can cause the valve to reopen and close again [7]. 

Restoration of sinus rhythm from atrial fibrillation can potentially cause reverse remodeling of the LA. This in turn will be beneficial for the structure of the mitral valvular apparatus which can improve the degree of MR. Treatment of MR itself has positive effects on augmentation in the stroke volume which will lead to further reduction in the size of LA and improvement in the severity of MR. Thus, anti-arrhythmic and ablative therapy would potentially be helpful in the prevention of the progression of MR. Additionally, the restoration of sinus rhythm can potentially save patients from invasive procedures for valve repair or replacement [13,19-22].

However, it has been shown that atrial fibrillation has an independent effect on the degree of MR. Gertz et al. showed that after successful ablation of atrial fibrillation, the severity of MR decreased significantly compared to the control group after one year of follow-up [9]. Additionally, they showed that the annular size, age of the patients, and the presence of persistent atrial fibrillation were independently associated with MR. In fact, maintaining sinus rhythm was more important than atrial remodeling. Bijl et al. showed that the improvement of MR was more prevalent in recipients of cardiac resynchronization therapy who were in sinus rhythm vs the patients who were in atrial fibrillation with the same degree of LV remodeling, which can be due to the greater LA volumes and mitral annular diameters in atrial fibrillation patients [11].

In the present case, the restoration of sinus rhythm was noted to have a positive effect on the severity of MR. This was evident even before the LA underwent reverse remodeling. This is in line with the findings of Gertz et al. and emphasizes the independent effect of the atrial fibrillation/flutter from LA remodeling on the degree of MR [7]. Although the mechanism was never fully investigated, it is possible that atrial flutter can also lead to the loss of the atrial 'kick' contraction although to a lesser extent than atrial fibrillation [18]. Another possibility is that ablation eliminates flutter waves, restoring coordinated atrial activity. This prevents early valve closure, allowing normal ventricular filling and synchronized valve function. Regaining atrial contraction after conversion to sinus rhythm would normalize annular size variation during the cardiac cycle [7,23]. 

The present study provides an example of an independent association between atrial flutter and MR severity, regardless of LA remodeling. It brings forward the idea that the reduction in MR before atrial remodeling could be the pathway toward treatment in atrial functional MR. It may offer some insight in answering the question “what comes first”: whether the atrial fibrillation and flutter cause significant MR with LA dilation or if an initial MR and LA remodeling trigger atrial fibrillation or flutter. Both situations may be plausible.

## Conclusions

This report presents a case of moderate-to-severe MR that improved shortly after successful ablation of atrial flutter with restoration of sinus rhythm; this improvement in the degree of MR took place even before the atrial remodeling happened. Although in the past few years, the concept of atrial functional MR has been introduced, its prevalence, mechanism, and risk factors are still not fully identified. Further studies with a larger patient population may help to understand the aspects and extent of atrial functional MR.
